# Mepiquat chloride promotes cotton lateral root formation by modulating plant hormone homeostasis

**DOI:** 10.1186/s12870-019-2176-1

**Published:** 2019-12-21

**Authors:** Qian Wu, Mingwei Du, Jie Wu, Ning Wang, Baomin Wang, Fangjun Li, Xiaoli Tian, Zhaohu Li

**Affiliations:** 1 College of Agronomy and Biotechnology, China Agricultural University, Beijing, 100193 China; 2 Institute of Agricultural Information, Jiangsu Academy of Agricultural Sciences, Nanjing, 210014 China; 3 Plant Phenomics Research Center, State Key Laboratory of Crop Genetics and Germplasm Enhancement, Nanjing Agricultural University, Nanjing, 210095 China; 4 Institute of Cotton Research of Chinese Academy of Agricultural Sciences, Anyang, 455000 Henan China

**Keywords:** Cotton, Mepiquat chloride (MC), Lateral root, Phytohormone, Transcriptome

## Abstract

**Background:**

Mepiquat chloride (MC), a plant growth regulator, enhances root growth by promoting lateral root formation in cotton. However, the underlying molecular mechanisms of this phenomenon is still unknown.

**Methods:**

In this study, we used 10 cotton (*Gossypium hirsutum Linn*.) cultivars to perform a seed treatment with MC to investigate lateral root formation, and selected a MC sensitive cotton cultivar for dynamic monitor of root growth and transcriptome analysis during lateral root development upon MC seed treatment.

**Results:**

The results showed that MC treated seeds promotes the lateral root formation in a dosage-depended manner and the effective promotion region is within 5 cm from the base of primary root. MC treated seeds induce endogenous auxin level by altering gene expression of both gibberellin (GA) biosynthesis and signaling and abscisic acid (ABA) signaling. Meanwhile, MC treated seeds differentially express genes involved in indole acetic acid (IAA) synthesis and transport. Furthermore, MC-induced IAA regulates the expression of genes related to cell cycle and division for lateral root development.

**Conclusions:**

Our data suggest that MC orchestrates GA and ABA metabolism and signaling, which further regulates auxin biosynthesis, transport, and signaling to promote the cell division responsible for lateral root formation.

## Background

The root system plays a pivotal role for plant growth by providing above ground mechanical support and controlling water and nutrient acquisition. Lateral roots, the major part of the root system in terms of root length and number, have crucial physiological capacities for water and nutrient uptake [1], and serve as the primary interface in response to heterogeneous soil environments [2]. Thus, the development of lateral roots has a decisive influence on both crop growth and harvest yield [3, 4].

The development of lateral roots is modulated by the regulatory networks integrating both genetic factors and endogenous hormones. Lateral root initiation originates from asymmetric cell division of xylem pole-pericycle cells induced by auxin-accumulation [5–10]. In plants, cell cycle and cell division progression are promoted by both the induction of positive regulators, such as cyclins (CYCs) and cyclin dependent kinases (CDKs), and the repression of negative regulators, including Kip-related proteins (KRPs) [11, 12]. The A-type CDKs and D-type CYCs complex play a critical role in cell cycle by regulating the G1-to-S transition. The G2-to-M checkpoint, regulating cell cycle progression to the mitotic phase, is mainly controlled by B-type CDKs and A- or B-type *CYCs* [13–15].

Auxin transport affects the asymmetric cell division [5, 6, 16–18]. Directional transport of auxin is controlled by the asymmetric distribution of auxin carriers, including the influx carrier, AUXIN TRANSPORTER PROTEIN 1 (AUX1) /like AUX1 (LAX), and the efflux carriers of the PIN-FORMED (PIN) family. AUX1 facilitates shoot-derived indole acetic acid (IAA) loading to the vascular transport system. LAXs mediates the formation of auxin gradient to generate acropetal auxin transport within inner tissues of the root apex and basipetal auxin transport within outer tissues of the root apex [19]. The *PINs* locates in a polar fashion, allowing directional auxin transport and the establishment of local auxin maxima and minima [5, 17, 20].

Auxin/Indole-3-Acetic Acid (Aux/IAAs), AUXIN RESPONSE FACTOR (ARF), and SMALL AUXIN UP RNA (SAUR) are key members responsible for auxin signal transduction. IAA14/SLR (SOLITARY ROOT)-ARF7-ARF19 and IAA12/BDL (BODENLOS)-ARF5 are important auxin signal modules involved in lateral root initiation [21–23]. Auxin-induced degradation of Aux/IAA proteins releases ARF7 and ARF19 transcription factors to activate downstream gene expression for lateral root initiation [23–25]. LBD (LATERAL ORGAN BOUNDARIES DOMAIN) transcription factor family, function in lateral root formation, is identified as one of the direct downstream components of ARF7 and ARF19 [22, 25–27].

In addition to auxin, other plant hormones, including gibberellin (GA), abscisic acid (ABA), ethylene, and jasmonate (JA), also have influence on lateral root formation in an auxin-dependent manner. GA negatively affects lateral root formation partially by the regulation of polar auxin transport in *Populus* [28]. The ABA receptor PYL8 promotes lateral root growth by enhancing auxin signaling [29, 30]. ABSCISIC ACID-INSENSITIVE 4 (ABI4) inhibits polar auxin transport by decreasing the expression of *PIN1* to influence lateral root formation [31]. Ethylene affects auxin signaling and transport to regulate root development [32, 33]. JA promotes lateral root formation by directly inducing the auxin biosynthesis and/or modulating *PIN2* accumulation on the plasma membrane [34, 35].

A plant growth regulator mepiquat chloride (MC), a gibberellin synthetic growth retardant, blocks the *ent-copalyl diphosphate synthase* (*CPS*) and *ent-kaurene synthase* (*KS*) in the early steps of GA metabolism [36]. MC is a water-soluble organic molecule and regulates the plant growth upon soaking seeds or spraying leaves with this molecule [37]. MC has been commonly used in cotton production to shorten internode elongation, reduce main stem nodes, and decrease plant height, leading to a more compact plant architecture [38–41]. Apart from plant canopy manipulation, MC also enhances root growth by increasing numbers of lateral roots. However, the underlying mechanism is largely unknown.

In this study, a MC sensitive cotton cultivar was selected to perform the transcriptome analysis by RNA-seq during lateral root development. We demonstrate that MC orchestrates GA and ABA metabolism, which further regulates auxin biosynthesis, transport, and signaling to control the cell division responsible for lateral root development.

## Results

### MC promotes the development of lateral roots in cotton seedlings

**Fig. 1:**
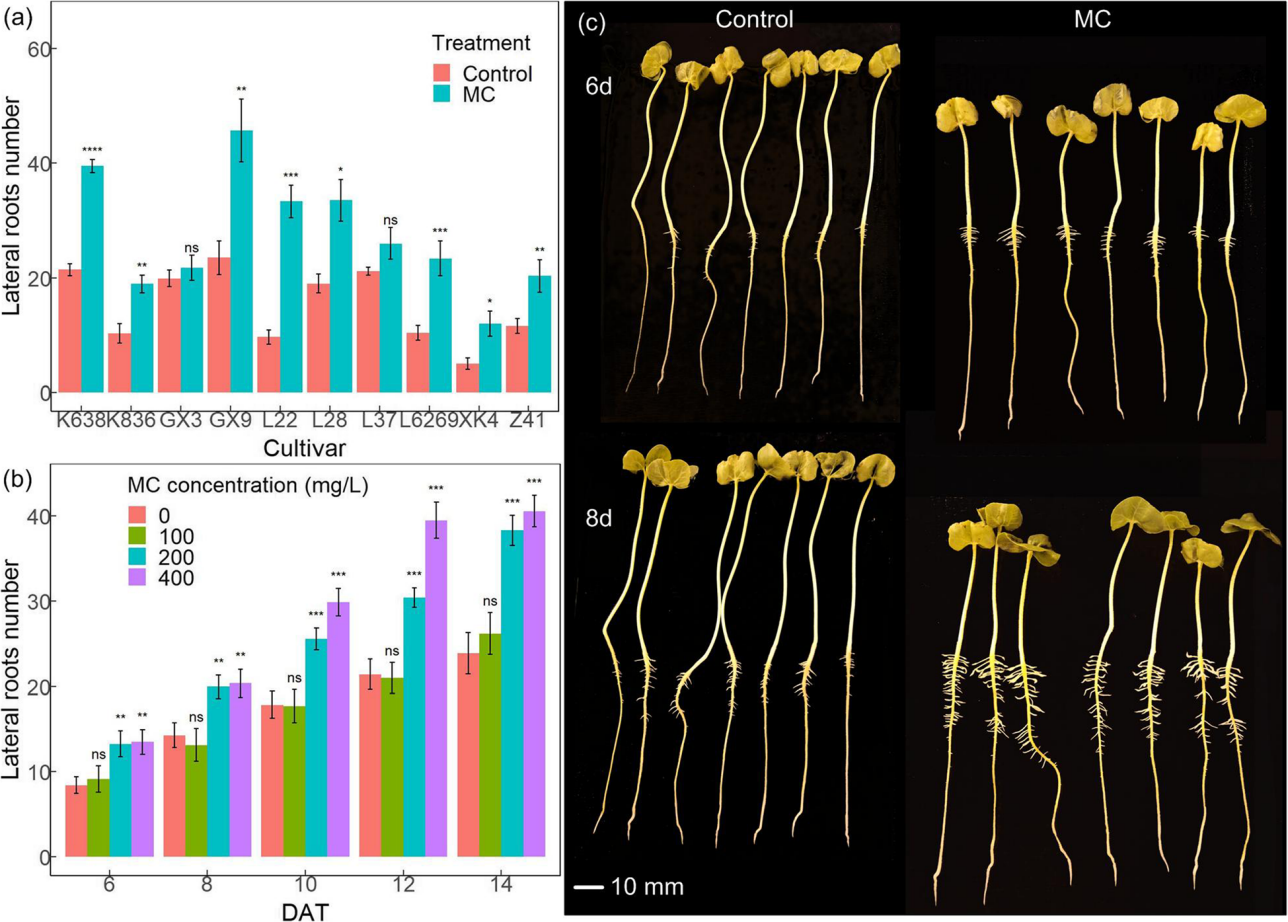
MC promotes lateral root development in cotton. **a** MC promotes lateral root development in different cotton cultivars. Seeds from 10 cotton cultivars were collected to detect their lateral root number 12 DAT of seeds soaked with deionised water (Control) or 400 mg/L MC for 12 h. Images were acquired to quantify the lateral root number. **b** MC promotes lateral root development in a dosage-depended manner. Cotton seeds of K638 were treated with 100 mg/L, 200 mg/L, and 400 mg/L MC. Images were acquired at indicated time points to quantify the lateral root number correspondingly. **c** Root phenotype of K638. Images were acquired at 6 and 8 days after MC treatment (DAT). Significant differences were assessed from three repeats by standard t-tests (*p < 0.05, **p < 0.01, ***p < 0.001, ****p < 0.0001). 3 replicates per treatment, 7 plants per replicate, a total of 21 seeds per treatment

To study the effects of MC on the lateral root formation, we pretreated cotton seeds with MC and found that seeds treated with MC significantly increased the lateral root number of the tested cotton cultivars, with the exception of GX3 and L37 (Fig. 1a). Among these cultivars, K638 had the most significant response to MC on lateral root formation and was selected for further analysis. Different concentrations of MC were applied to detect lateral root formation of K638. The results showed that the induction of MC on lateral root formation was dosage dependent. The number of lateral roots had no obvious increase with 100 mg/L MC treatment compared with H_2_O treatment. While, 28.5–37.7% and 30.1–45.8% induction of lateral root number were observed with 200 mg/L and 400 mg/L MC treatment compared with H_2_O treatment (Fig. 1b). After 8 days of MC treatment, K638 developed more and longer lateral roots compared with the control (Fig. 1c). The results showed that soaking seeds with MC promotes lateral root formation in cotton seedlings in a dosage dependent manner.

### Dynamic monitoring of MC effects on root growth

**Fig. 2:**
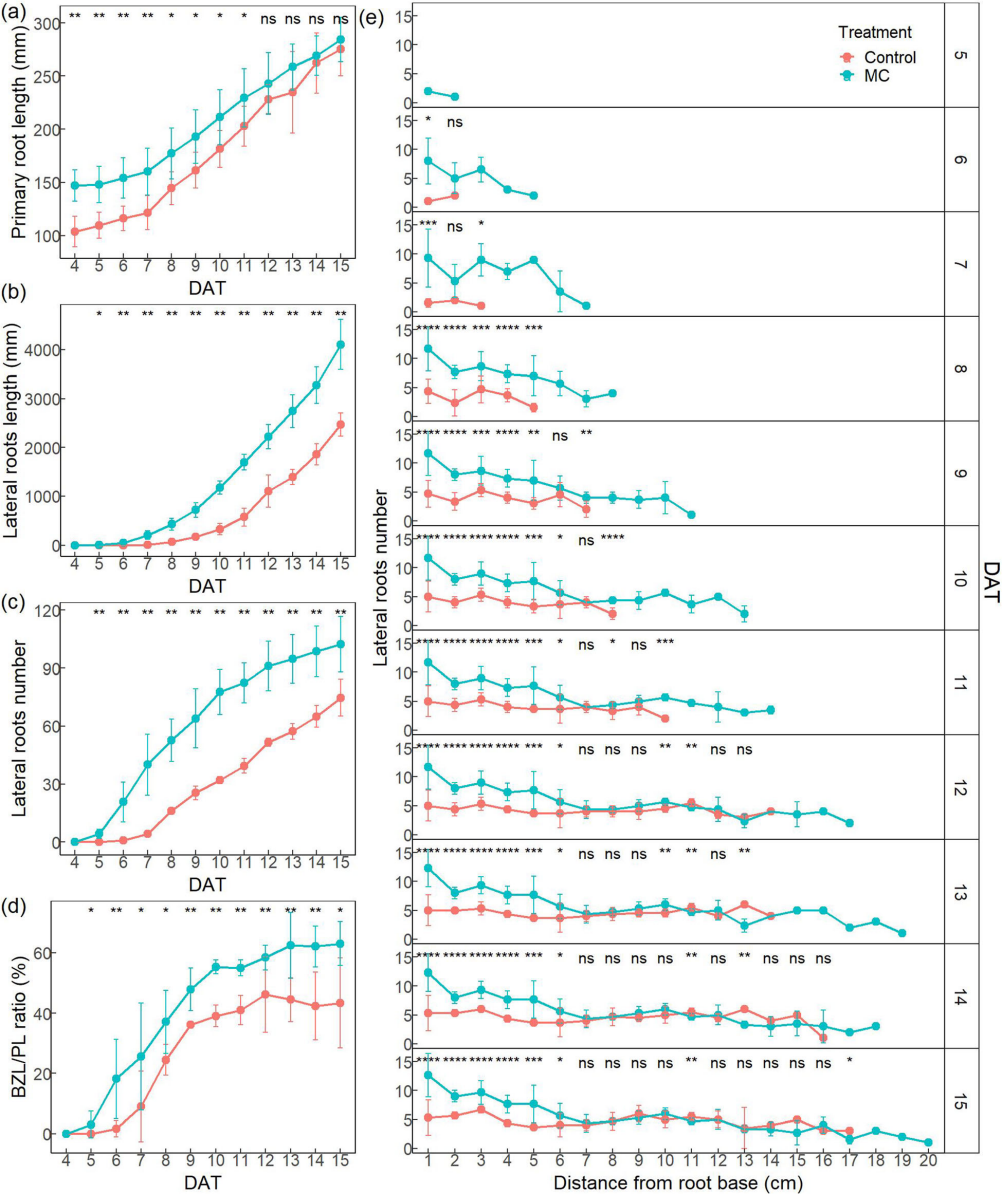
MC promotes dynamic growth of cotton roots. MC treated seeds increases the primary root length (**a**), the total length (**b**) and total number (**c**) of lateral roots, the BZL/PL ratio (**d**), and the lateral root number within 5 cm from the base of primary roots (**e**). Cotton seeds of K638 were treated with deionised water (Control) or 400 mg/L MC for 12 h. The dynamic growth of root systems were monitored by using RhizoChamber-Monitor and were quantified by using the customized image processing software. Significant differences were assessed by standard t-tests (*p < 0.05, **p < 0.01, ***p < 0.001, ****p < 0.0001). 3 replicates per treatment, 3 plants per replicate, a total of 9 seeds per treatment

To study the dynamic effects of MC on root growth, the cotton seedlings of K638 were cultured using the customized high-throughput robotic platform RhizoChamber-Monitor [42] to monitor the dynamic growth of root systems. Primary root length was significantly increased compared to Control (the deionised water treatment). The primary root length was higher for MC treatment(Fig. 2a). For the lateral roots, both the total root length and root number of MC treatment were significantly more than that of Control (Fig. 2b, c). The increase rate of lateral root length upon MC treatment was significantly more than that of Control at seven DAT, while the increase rate of lateral root number was significantly higher for MC treatment from the day 5 (Fig. 2b, c). In addition, the BZL/PL ratio (Branching zone length /Primary root length) of MC treatment was significantly more than that of Control (Fig. 2d), and the increasing rate of BZL/PL ratio was higher before nine DAT.

In order to further identify the origin of lateral root induced by MC treatment in the primary root, the variations of lateral root number along 1 cm sections from root base to root tip in primary roots were quantified during the dynamic growth of root systems. The number of lateral roots was significantly more for MC treatment within 5 cm from the base of primary roots (Fig. 2e). In addition, the lateral roots emerged much earlier with MC treatment than that of Control. The promotion effect of MC on lateral root formation was obvious before seven DAT. Together, soaking seeds with MC promotes the lateral root formation and advances the emerge-time of lateral roots.

### Transcriptome analysis of MC-treated cotton seeds

To explore the dynamics of gene expression during cotton root development upon MC treatment, we performed RNA-seq analyses in cotton roots. An overview of the sequence assembly after Illumina sequencing was shown in Additional file 1: Table S1. Q20 and Q30 were above 96 and 92%. The average error rate was less than 0.02%. The percentage of low (FPKMs in the interval 1–3), medium (FPKMs in the interval 3–15), and high (FPKMs beyond 15) level expressed genes in control was 15, 24.8, and 15.5%, respectively (Additional file 2: Table S2). The relationship of transcriptome samples for Control and MC treatment at three time points were assessed by a principal component analysis (PCA) and hierarchical clustering (Additional file 6: Figure S1). The data indicate that three biological replicates of each treatment have strong correlation.

**Fig. 3:**
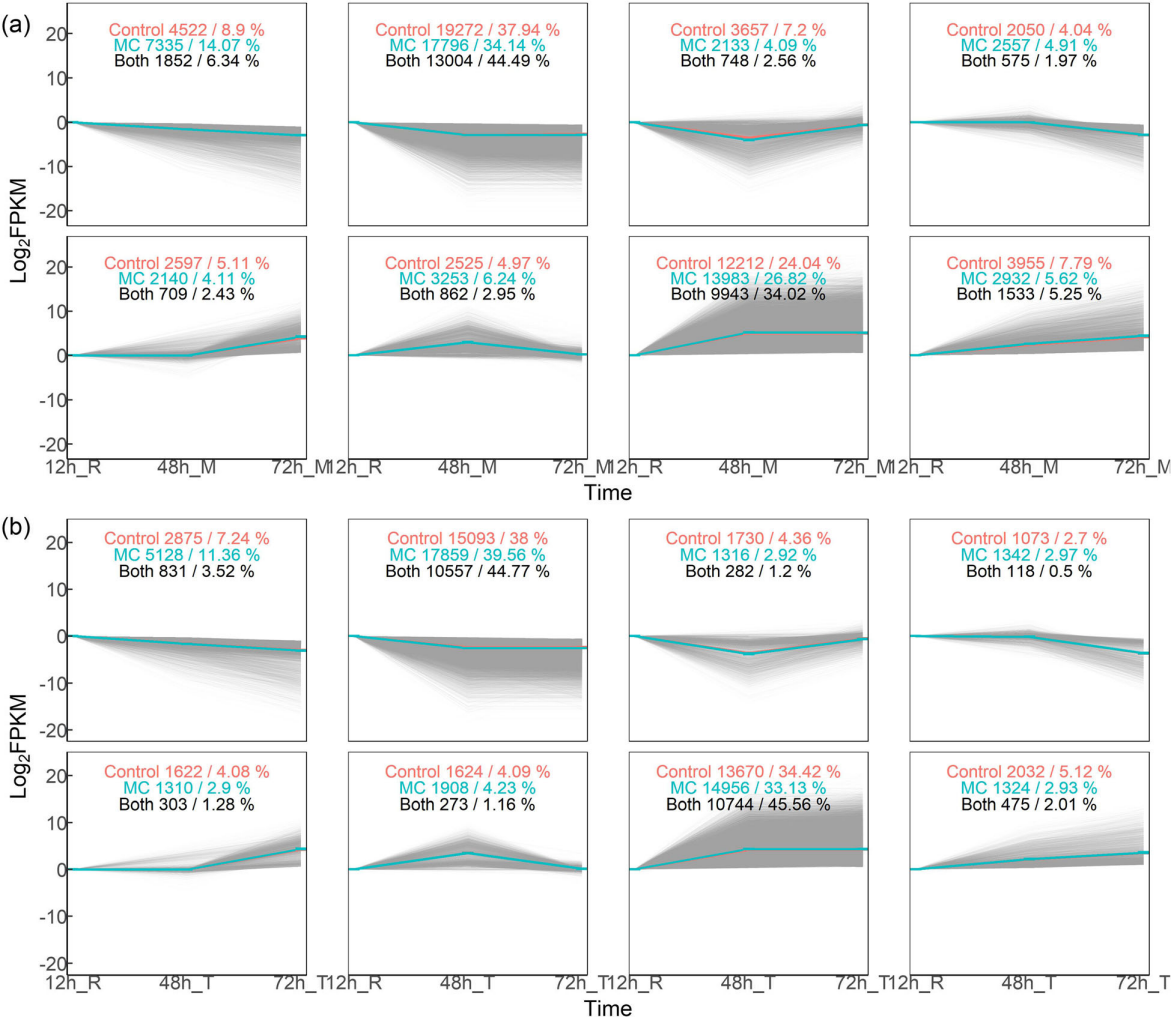
The dynamics of gene expression during cotton root development. K-means clustering showing eight patterns of gene expression along three time points for the root middle region (**a**) and root tip (**b**), respectively. Cotton seeds of K638 were treated with deionised water (Control) or 400 mg/L MC for 12 h. The roots were sampled at 12, 48, and 72 h after treatment and subjected to RNA-seq analyses. “R” indicates the whole root, “M” indicates the root middle region (4 to 20–40 mm from the root tip), “T” indicates the root tip region (0 to 4 mm from the root tip). Numbers in red and green indicate the genes following the respective pattern for Control and MC treatment, respectively. Numbers in black indicate genes displaying identical patterns in both treatments. 3 biological replicates per treatment, 30 roots per replicate

Eight patterns of gene expression along the three time points were identified in root tip and root middle region for CK and MC treatment by K-means clustering (Fig. 3). The similarity of gene expression levels was analyzed according to Euclidean distance. At root middle region, 50,790 and 52,129 genes were assigned to eight clusters (K1 to K8) for Control (red numbers) and MC treatments (green numbers), respectively (Fig. 3a). In total, 29,226 genes (56.1%/57.5% in MC/Control treatment) exhibited the same expression pattern in both treatments (black numbers). At root tip, 39,719 and 45,143 genes were assigned to eight clusters for Control and MC treatments, respectively (Fig. 3b). In total, 23,583 genes (51.9%/59.3% in MC/Control treatment) exhibited the same expression pattern in both treatments. Taking together, MC treated seeds change the transcriptome dynamics during cotton root development.

**Fig. 4:**
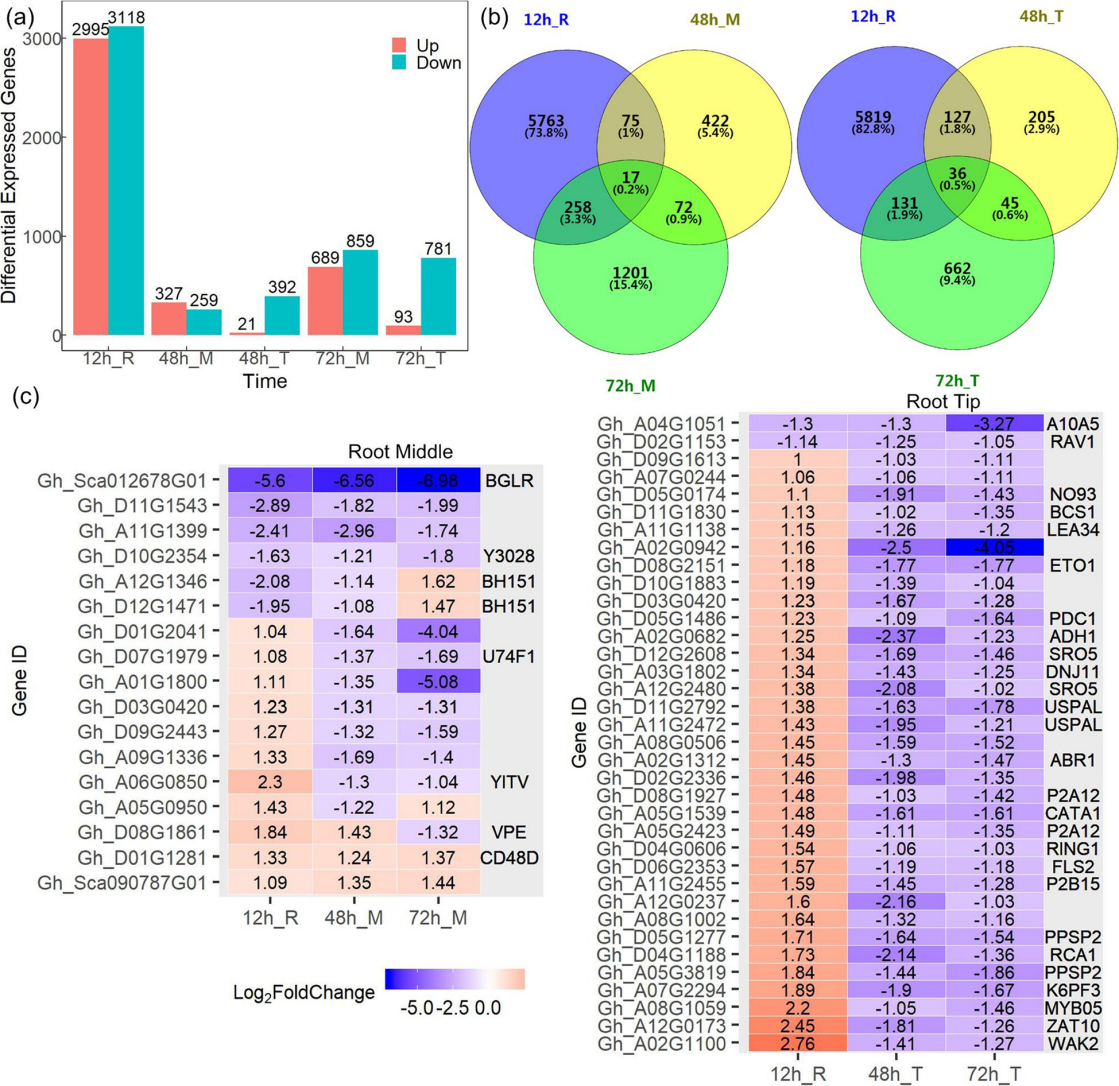
DEGs regulated by MC during root development in cotton. **a** The total number of up- and down-regulated DEGs in root middle region and root tip at three time points. **b** Venn diagram displaying DEGs and common genes upon soaking-seed treatment at three time points in root middle region and root tip. **c** Genes differentially expressed at all three time points in root middle region and root tip. Cotton seeds of K638 were treated with deionised water (Control) or 400 mg/L MC soaking-seed for 12 h. The roots sampled at 12, 48, and 72 h after treatment were subjected to RNA-seq analyses. “R” indicates the whole root, “M” indicates the root middle region (4 to 20–40 mm from the root tip), and “T” indicates the root tip region (0 to 4 mm from the root tip). The DEGs were controlled by FDR < 5% and |log_2_Fc| ≥ 1. Values in boxes are log_2_Fc. 3 biological replicates per treatment, 30 roots per replicate

Differentially expressed genes (DEGs) were further determined between Control and MC soaking-seed treatment (Fig. 4). Overall 6113 DEGs (FDR < 5%, |log_2_Fc| ≥ 1) in whole root at 12 h (12 h_R), 586 DEGs in root middle region (48 h_M), 413 DEGs in root tip at 48 h (48 h_T), 1548 DEGs in root middle region (72 h_M), and 874 DEGs in root tip at 72 h (72 h_T) were identified after MC treatment (Fig. 4a). Upon DPC treatment, 17 (0.2%) DEGs were observed at all three time points; whereas 7386 (94.6%) genes were differentially expressed at only one time point (Fig. 4b). In root tip, 36 (0.5%) DEGs were observed at all three time points; whereas 6686 (95.2%) genes were differentially expressed at only one time point (Fig. 4b). Among the common DEGs (FDR < 5%, |log_2_Fc| ≥ 1) at all three time points in root middle region (Fig. 4c), four genes were down-regulated, and two genes were up-regulated, including *CD48D* gene encoding a cell division control protein; two bHLH transcription factor genes (*BH151*) that modulate the balance between cellular proliferation and differentiation in root growth [43], were down-regulated at 12 h and 48 h, while up-regulated at 72 h. For the root tip (Fig. 4c), genes encoding auxin-induced protein A10A5 and two transcription factors AP2/ERF and B3 domain-containing RAV1 were down-regulated at all three time points; 34 genes were up-regulated at 12 h and down-regulated at 48 h and 72 h, including ethylene-responsive transcription factor *ABR1*, a negative regulator in ABA signaling pathway, myb-related transcription factor *MYB05*, ethylene-overproduction protein gene *ETO1*, and wall-associated receptor kinase gene *WAK2*. Most of these DEGs were plant hormones-related genes that are involved in root development.

### GO and KEGG analysis

The DEGs were assigned to different functional categories using GOseq R package. The DEGs between Control and MC treatments (q-value< 0.05) were categorized into 54 functional groups. For biological process, 407, 405, 136, and 130 DEGs were enriched for the categories ‘R biosynthetic process’ (GO:0032774), ‘transcription, D-dependent’ (GO:0006351), ‘electron transport’ (GO:0006118), and ‘response to chemical stimulus’ (GO:0042221) (Additional file 3: Table S3). For molecular function, 336, 64, and 64 DEGs were enriched for the categories ‘oxidoreductase activity’ (GO:0016491), ‘sequence-specific D binding transcription factor activity’(GO:0003700), and ‘nucleic acid binding transcription factor activity’(GO:0001071) (Additional file 3: Table S3). The functional annotation and GO enrichment of the down-regulated and up-regulated DEGs at each time point of root middle region and root tip were shown in Additional file 7: Figure S2.

To identify the biological pathways involved in lateral root development upon MC treatment, we used KOBAS software to test the statistical enrichment of DEGs in KEGG pathways. Two thousand four hundred eighty-six DEGs between Control and MC treatment were assigned to 35 KEGG pathways (Additional file 8: Figure S3). Among them, 696 DEGs were assigned to metabolic pathways, 451 DEGs were assigned to biosynthesis of secondary metabolites, and 312 DEGs were assigned to plant hormone signal transduction.

### MC modulates the expression profile and accumulation of multiple plant hormones

**Fig. 5:**
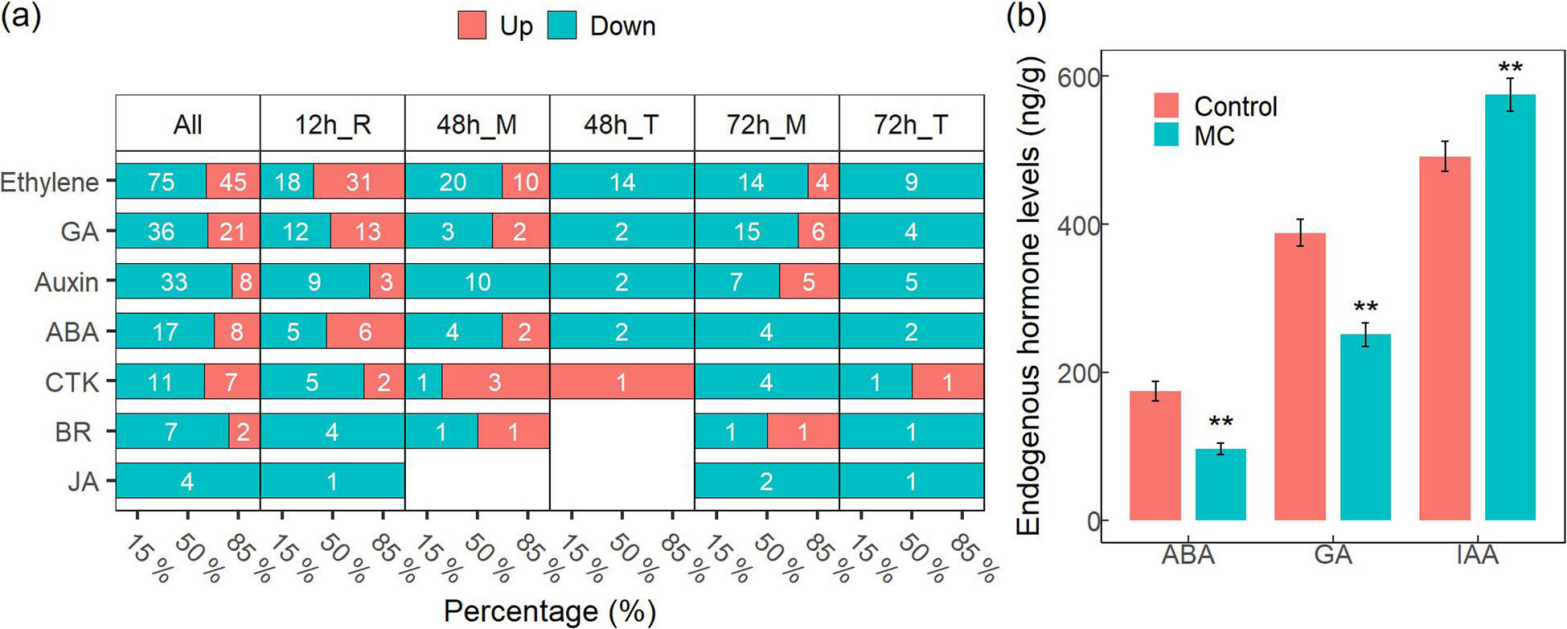
MC modulates the expression profile and accumulation of multiple plant hormones. **a** MC modulates the differentially expressed profile in hormone metabolism, transport, and signaling. The DEGs were controlled by FDR < 5% and |log_2_Fc| ≥ 1. The percentage represent the ratio of up−/down-regulated genes number to the total number of regulated-genes. **b** MC induces the IAA level and reduces ABA and GA levels in cotton roots at five DAT. Cotton seeds of K638 were treated with deionised water (Control) or 400 mg/L MC for 12 h. Segments of approximately 2 cm root apex were cut at five DAT and endogenous free IAA, GA, and ABA were measured. Significant differences were assessed by standard t-tests (**p < 0.01). 3 biological replicates per treatment, 30 roots per replicate

Phytohormones play a key role in plant growth and are closely related to root development. Next, we investigated the enriched functional category of hormone metabolism upon MC seed treatment and found that 274 DEGs were involved in hormone metabolism, signaling, or response (Fig. 5a). These DEGs were mainly related to ethylene (120 genes, 43.8%), GA (57 genes, 20.8%), auxin (41 genes, 15%), ABA (25 genes, 9.1%), and CTK (18 genes, 6.6%). 89% DEGs were related to ethylene and belonged to the *ERF/EF* transcription factor family. In addition, nine DEGs were related to brassinosteroid (BR) and four DEGs were related to JA were observed.

To further confirm the involvement of these hormones, we detected the concentrations of GA, ABA, and IAA in cotton roots upon MC seed treatment. The data showed that treatment of MC induced IAA levels, but reduced GA and ABA levels in cotton roots at 5 days after treatment (DAT5) (Fig. 5b). The data indicates that MC could orchestrate hormone homeostasis.

### MC treatment alters the expression of GA-, ethylene-, and ABA -related genes

**Fig. 6:**
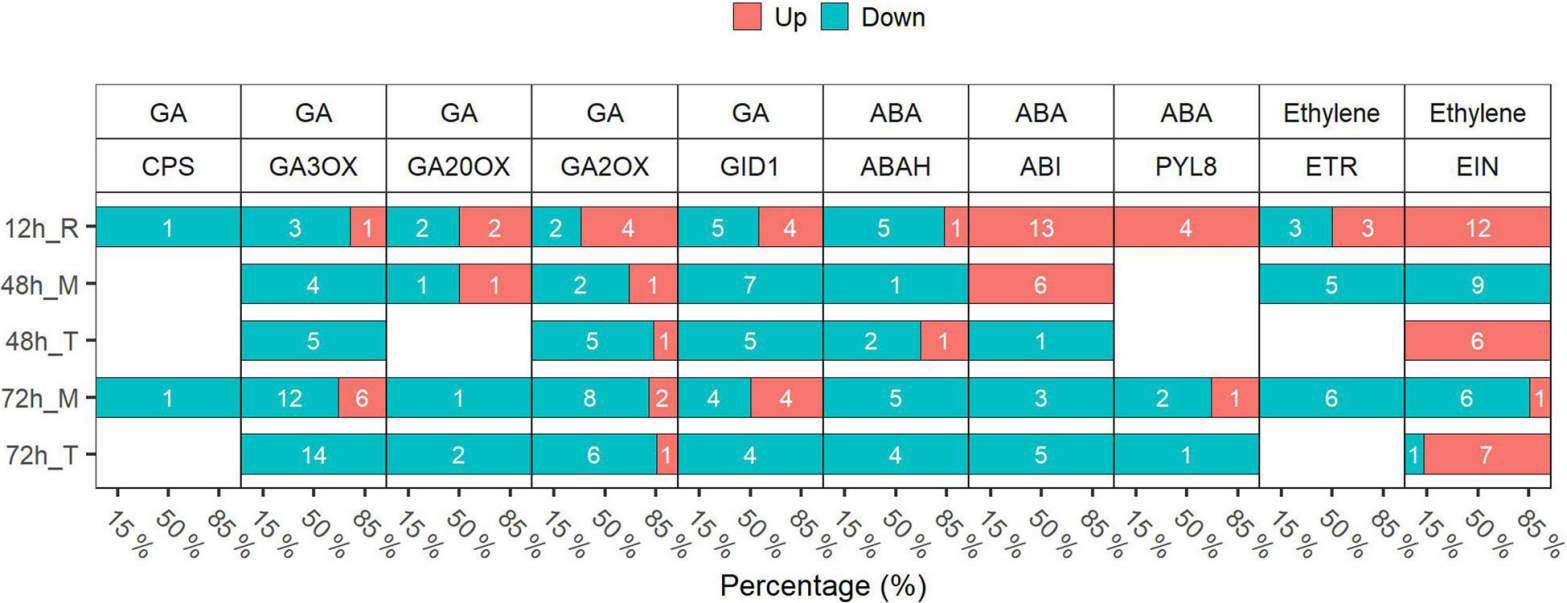
MC regulates the expression of GA-, ethylene-, and ABA -related genes. The DEGs were controlled by FDR < 5%. Cotton seeds of K638 were treated with deionised water (Control) or 400 mg/L MC for 12 h. The roots sampled at 12, 48, and 72 h after treatment were subjected to RNA-seq analyses. “R” indicates the whole root, “M” indicates the root middle region (4 to 20–40 mm from the root tip), and “T” indicates the root tip region (0 to 4 mm from the root tip). White digit indicated the gene number. The percentage represent the ratio of up−/down-regulated genes number to the total number of regulated-genes. 3 biological replicates per treatment, 30 roots per replicate

Plant hormones have important influence on lateral root formation [30, 33, 35]. Specifically, GA-, ethylene-, and ABA-related DEGs were identified at each time points (the genes were controlled by FDR < 5%) (Fig. 6). GA biosynthesis genes *CPS*, *GA20OX*, and *GA3OX* were down-regulated by MC treatment. While *GA2OX*, a gene involved in GA catabolism, was up-regulated at 12 h. The GA receptor GIBBERELLIN INSENSITIVE DWARF1*, GID1* was down-regulated. GA plays negative roles in lateral root formation. Consistently, our results showed that MC represses GA biosynthesis and signaling during lateral root formation. We also found that ethylene receptor *ETRs* and Ethylene-insensitive protein (*EIN*) family genes were down-regulated in the root middle region at 48 h and 72 h. Moreover, we observed that the *ABAH* gene family, involved in the oxidative degradation of ABA, was down-regulated. While, ABSCISIC ACID-INSENSITIVE3/4/5 (*ABI3/4/5*) and ABA receptor PYL8, both which function in ABA signaling, were up-regulated at 12 h.

### MC modulates the expression of auxin-related genes

**Fig. 7:**
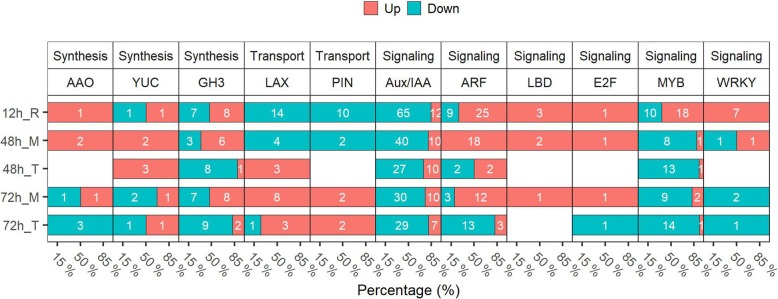
MC modulates the expression of the auxin-related genes. The DEGs were controlled by FDR < 5%. Cotton seeds of K638 were treated with deionised water (Control) or 400 mg/L MC for 12 h. The roots sampled at 12, 48, and 72 h after treatment were subjected to RNA-seq analyses. “R” indicates the whole root, “M” indicates the root middle region (4 to 20–40 mm from the root tip), and “T” indicates the root tip region (0 to 4 mm from the root tip). White digit indicated the gene number. The percentage represent the ratio of up−/down-regulated genes number to the total number of regulated-genes. 3 biological replicates per treatment, 30 roots per replicate

Auxin is the major hormone to control lateral root development. Auxin-related DEGs that belonged to different families were identified at each time points (Fig. 7, Additional file 4: Table S4). For the auxin synthesis DEGs, *AAO2*, encoding the enzyme for oxidizing indole-3-acetaldehyde to IAA, was up-regulated at 12 h and 48 h in the root middle region; YUC family genes, which encode flavin monooxygenase-like proteins that catalyze a rate-limiting step in IAA biosynthesis, were up-regulated at 48 h, indicating that MC could induce auxin biosynthesis. GH3 family genes, which encode IAA-amido synthetases, were mostly down-regulated at 48 h and 72 h in root tip. Meanwhile, auxin transport genes, like LAX and PIN family genes were down-regulated at 12 h and 48 h, but up-regulated at 72 h, indicating that MC may gradually regulate auxin transport to promote lateral root formation. DEGs related to auxin signaling, like negative regulator Aux/IAA family genes, were down-regulated by MC treatment. Moreover, over half of the *ARF* family genes were up-regulated at 12 h in root and at 48 h and 72 h in the root middle region. The *LBDs* and *E2Fs* transcription factors, two downstream components of *ARF,* were up-regulated at 12 h in root and at 48 h and 72 h in the root middle region. In addition, some auxin-related transcription factors, like WRKY and MYB, were up-regulated at 12 h, suggesting that the auxin signaling was enhanced upon MC treatment in the lateral root initiation zone.

### MC promotes lateral root development through regulating cell cycle and division

**Fig. 8:**
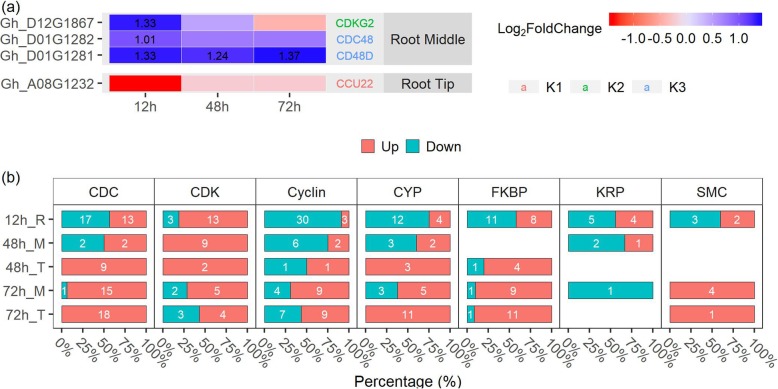
MC regulates the expression of cell cycle and cell division related genes. **a** Expression dynamics of genes differentially expressed at all three time points. DEGs satisfy |log_2_Fc| ≥ 1 at least one time point were collected. K1:down-down; K2: up-down; K3:up-up. Values in boxes are log_2_Fc (show |log_2_Fc| ≥ 1 only). **b** MC overall induces genes in cell cycle and cell division. White digit indicated the gene number. The percentage represent the ratio of up−/down-regulated genes number to the total number of regulated-genes. Cotton seeds of K638 were treated with deionised water (Control) or 400 mg/L MC for 12 h. The roots sampled at 12, 48, and 72 h after treatment were subjected to RNA-seq analyses. “R” indicates the whole root, “M” indicates the root middle region (4 to 20–40 mm from the root tip), “T” indicates the root tip region (0 to 4 mm from the root tip). 3 biological replicates per treatment, 30 roots per replicate

Cell proliferation and differentiation regulate lateral root initiation. Genes known to be involved in cell cycle and cell division were identified at root middle region and root tip, respectively (Fig. 8a). At the root middle region, two cell division cycle protein gene family, *CDC48* and *CD48D*, were up-regulated at all three time points; one cyclin-dependent kinase gene, *CDKG2*, was also up-regulated by MC treatment. Specifically, cell cycle and cell division related DEGs that belonged to different families were identified at each time points (Fig. 8b). Most of the *CYCs* family genes, which are involved in the control of the cell cycle at the G1/S (start) transition, were up-regulated at 72 h. Majority of *CDK* genes were up-regulated at 72 h and *KRP* genes were down-regulated at 12 h by MC treatment. Most *CDC* family genes, peptidyl-prolyl isomerase (PPIase) family genes (*CYP* and *FKBP*), and the structural maintenance of chromosomes protein gene (*SMC*) was up-regulated at 72 h. The results suggest that MC promotes lateral root formation by regulating genes related to cell cycle and cell division.

## Discussion

### MC promotes cotton lateral root formation

MC is a commonly used plant growth regulator in cotton production and enhances root growth by increasing the number of lateral roots and root biomass [37]. However, the underlying molecular mechanism on how MC promotes lateral root initiation in cotton is unclear. Our studies show that soaking seeds with MC significantly promotes the cotton lateral root formation in a dosage-depended manner (Fig. 1a, b). With RNA-Seq analysis, large number of DEGs, especially genes related to hormone metabolism and signaling, were determined during lateral root formation with MC treated seeds.

### MC mediates the crosstalk among GA, ABA, and IAA during lateral root formation

Inhibitors of GAs biosynthesis, such as paclobutrazol, can enhance lateral root formation in tomato (*Solanum lycopersicum*) [44], pepper (*Capsicum chinense*) [45], and several tree species [46]. Mutants defective in GA biosynthesis or signaling were found to have stimulated lateral root formation [28, 44, 47]. MC promotes lateral root development likely via the regulation of endogenous gibberellin. Here, the RNA-seq results showed that MC down-regulates GA biosynthesis genes, like *CPS*, *GA20oxs*, and *GA3ox*, while up-regulates GA catabolism gene *GA2ox* (Fig. 6 and Additional file 9: Figure S4). Consistently, the GA levels in root was significantly lower for MC treated seeds compared with the control (Fig. 5b).

The crosstalk of GA and other hormones, including auxin and ABA, play important roles in modulation of lateral root development [28]. GA receptor GID1 induces the degradation of DELLA proteins, and further regulates the expression of XERICO, an inducer of ABA biosynthesis [35, 48]. Thus, endogenous ABA levels may be increased by MC treatment at the stage of seed development. GA negatively affects lateral root formation partially by the regulation of polar auxin transport in *Populus* [28]. Our results show that MC treatment significantly increases auxin levels and reduces ABA levels in root tissues at five DAT (Fig. 5b), consistent with the result that GA affects lateral root formation through auxin-mediated pathway or direct down-regulation of ABA biosynthesis [28].

Auxin is known to be the critical phytohormone involved in regulating lateral root development [23]. Auxin maxima in the lateral root initiation zone is a key factor for the formation of lateral root primordial, which is maintained via the activation of auxin biosynthesis and transport [5, 6, 16–18]. ABA could influence lateral root formation by regulating auxin signaling and transport [29–31]. However, there are contradictory understandings of ABA effects on lateral root formation. The ABA receptor *PYL8* promotes lateral root growth by the activation of *MYB44* to induce auxin signaling [29, 30]. In contrast, Shkolnik-Inbar and Bar-Zvi reported that ABA inhibits lateral root formation by regulating auxin transport [31]. *ABI4* inhibits polar auxin transport in the root by decreasing the expression of *PIN1*; and the expression of *ABI4* is enhanced by ABA and repressed by auxin. In our study, *PYL8* and *MYB44* were up-regulated at 12 h (Figs 6, 7 and Additional file 9: Figure S4); *ABI5* was down-regulated. Meanwhile, *PINs* were up-regulated at 72 h (Fig. 7). Thus, it is likely that MC treatment enhances auxin signaling via up-regulating ABA receptor *PYL8*, and enhances auxin signaling via down-regulating *ABIs* to promote lateral root growth. Negi et al. [32] reported that *etr1* or *ein2* mutants in ethylene signaling increase lateral root formation via the crosstalk with auxin signaling and transport pathways. Consistently, our data show that ETR family genes were down-regulated at 48 h and 72 h. Most EIN family genes were down-regulated in the root middle region (Fig. 6). Taken together, these results provide evidence that MC acts as an important regulator of lateral root development in a hormone crosstalk manner.

### MC directly regulates auxin metabolic, transport, and signaling

Auxin biosynthesis mediated by YUC or TAA promotes lateral root formation [49]. Overexpression of *YUC1/6* and *TAA1* increases IAA concentrations along with enhanced lateral root formation in *Arabidopsis thaliana* [49]. The auxin efflux regulators PIN and auxin influx carrier AUX1 mediate auxin transport in lateral root formation [5]. In our study, YUC family genes were up regulated by MC treatment at 48 h (Fig. 7). In addition, most auxin influx carriers LAX family genes and auxin efflux carriers PIN family genes were up-regulated at 72 h after MC treatment (Fig. 7 and Additional file 11: Figure S6). The IAA levels in roots was significantly higher for MC treated seeds (Fig. 5b). Thus, MC treated seeds could promote lateral root formation by directly regulating the expression of auxin biosynthesis and transport genes.

As the most important members of auxin signal transduction, *Aux/IAAs* family genes play a role in lateral root formation [21, 50–52]. Several Aux/IAA-ARF modules have been implicated in driving lateral root formation [25]. The IAA28-ARF5/6/7/19 module is specific for priming cell specification [6, 53]. The IAA14 -ARF7/19 module and IAA12-ARF5 module are specific for lateral root initiation and patterning [21–23]. The repression of *IAA12* and *IAA14* induce *ARF5* and *ARF7/19* gene expression to activate the cell cycle and form a lateral root primordial [25]. In our study, most Aux/IAA family genes were down-regulated by MC treatment, including *IAA14*, *IAA18*, and *AUX28* (Fig. 7 and Additional file 9: Figure S4, Additional file 11: Figure S6). However, some ARF family genes, like *ARF5/6/7* and *ARF19* in the root middle region were up-regulated (Fig. 7 and Additional file 9: Figure S4, Additional file 11: Figure S6). The *LBD* transcription factor family is a downstream component of *ARF7* and *ARF19*, and is involved in various root-related developmental processes [54–56]. *LBD16*, *LBD18*, *LBD29*, and *LBD33* have the cooperative regulation on the initiation and emergence of lateral root in Arabidopsis [26, 57, 58]. In this study, *LBD16* and *LBD18* were up-regulated by MC treatment (Fig. 7 and Additional file 9: Figure S4, Additional file 11: Figure S6). *LBD18/LBD33* dimer could further regulates *E2Fa* and *E2Fb* gene expression which are two transcriptional activator in cell cycle to stimulate cell entry into both S- and M-phases [27, 59, 60]. Together, the data indicate that MC activates auxin signaling mainly via the induction of auxin-related transcription factors during lateral root formation.

### MC induces genes in cell cycle during lateral root formation

The formation of lateral root is controlled by auxin-mediated cell cycle and cell division. The auxin-dependent cell cycle is mainly controlled by CDKA and CDKB [61]. The activity of CDK is largely determined by association with different cyclin partner CYCs [11, 15, 62]. Auxin and CTK increase the expression level of *CYCD* to activate CDKA, indicating their important role for mitotic activity in cell division [63–65]. In our study, most *CYCs* and *CDK* family genes, including *CYCA/B* and *CDKB*, were up-regulated at 72 h after MC treatment. KRP, as a negative regulator at early lateral root initiation by blocking the G1-to-S transition, is transcriptionally regulated by auxin [11, 62]. Here, we showed that KRP was down-regulated by MC treatment of seeds (Fig. 8b and Additional file 10: Figure S5). *CDC45* is required for initiation of chromosomal DNA replication. It acts at the origin of replication and in minichromosome maintenance [66, 67]. The peptidyl-prolyl isomerase (PPIase) family genes (CYP and FKBP) are essential for regulation of mitosis and cell growth [68]. The chromosomal structure maintains SMC is involved in cell cycle and DNA repair progression [69]. In this study, CDCs, FKBP, CYP, and SMC were all up-regulated at 72 h after MC treatment (Fig. 8b and Additional file 10: Figure S5), suggesting that MC induces lateral root formation by transcriptional regulation of the cell cycle.

## Conclusion

**Fig. 9:**
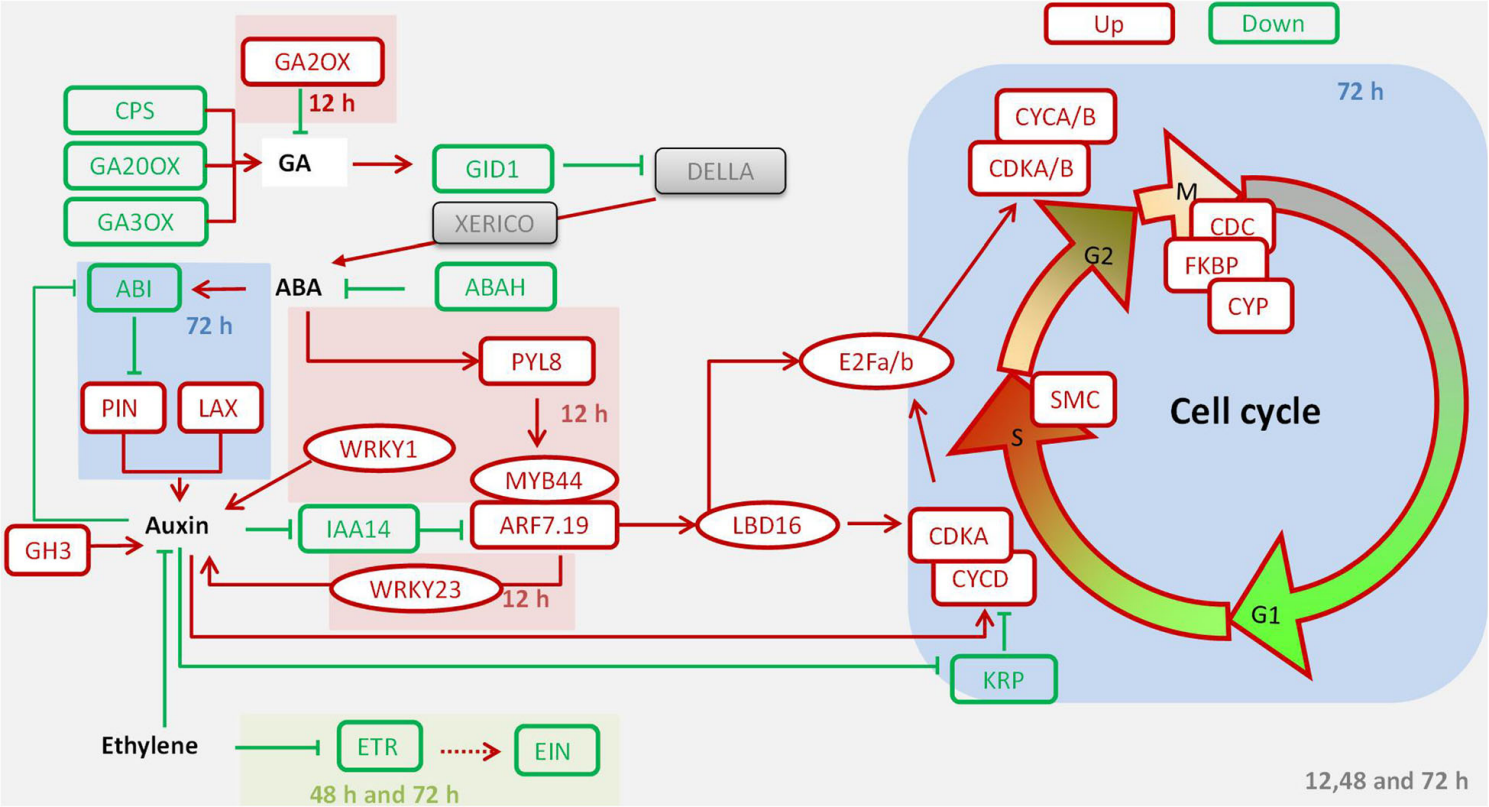
A model of MC-regulated lateral root formation in cotton. MC inhibits GA biosynthesis by down-regulating *CPS, GA20oxs,* and *GA3ox* and up-regulating *GA2ox*. MC down-regulates *GID1* to activate *DELLA* and *XERICO*, the inducer in ABA biosynthesis; furthermore, down-regulated ABAH decreases the oxidative degradation of ABA, promoting ABA accumulation. Meanwhile, MC activates ABA signaling by up-regulating its receptor *PYL8*, which induces the expression of *MYB44* and *ARF7*, likely promoting the expression of *WRKY23* to activate auxin signaling. Auxin transport genes *LAX* and *PIN* are up-regulated to regulate auxin maxima in the lateral root initiation zone. Up-regulated *YUC* and *AAO* increase auxin biosynthesis. Auxin-induced degradation of *IAA14* de-represses *ARF7/19* to induce *LBD16/18,* which is responsible for the activation of *E2Fa/b* to promote the first asymmetric cell divisions. Moreover, auxin increases the expression of *CYCD* and *CDKA* to regulate the G1-to-S transition, *CDKA/B* and *CYCA/B* to regulate the G2-to-M transition, and *CDCs, FKBP,* and *CYP* to regulate mitosis. In conclusion, our data suggest that MC orchestrates GA and ABA metabolism, which further regulates auxin signaling, transport, and biosynthesis to control the cell division responsible for lateral root formation

In general, the promoting effect of MC on lateral root formation is dosage dependent and shows a limited, effective duration and location in cotton roots. Here, we identified that MC soaking-seed inhibits GA biosynthesis and reduces GA level by regulating the genes in GA synthesis and signaling. The genes in ABA signaling are further affected because of the MC treated seeds also affect auxin signaling and transport by regulating genes involved in auxin biosynthesis, transport, and signaling, and the cell cycle and division process, have transcriptional change and were selected for further confirmation by qRT-PCR (Additional file 11: Figure S6). These results confirm that MC orchestrates phytohormone-dependent transcriptional networks during lateral root initiation (Fig. 9). Ethylene alters rooting by modulation of polar auxin transport upon MC treatment. Moreover, MC affects the auxin biosynthesis and auxin transport by regulating the expression of *YUC*, *AAO*, *PIN* and *LAX* genes, suggesting that MC affects lateral root formation by promoting the cooperation of auxin with other phytohormones. Furthermore, with the activation of auxin signaling, the downstream targets, *LBDs* and *E2Fs*, were regulated to control the cell cycle progression. The repression of *KRPs*, and the induction of *CYCs*, *CDKs*, *CDCs*, *FKBP*, *CYP*, and *SMC*, activate the cell cycle and promote cell division progression. The results indicate that MC promotes lateral root formation likely through the induction of auxin metabolism and signaling to active cell cycle and division processes via regulating hormone homeostasis.

## Materials and methods

### Plant materials, experimental set-up and growth conditions

Ten cotton (*Gossypium hirsutum Linn.*) cultivars K638, K836, Lu22 (L22), Lu28 (L28), Lu37 (L37), Lu6269 (L6269), Guoxin3 (GX3), Guoxin9 (GX9), Xinkang4 (XK4), and Zhong41 (Z41) were used in this study (provided by Hebei Guoxin Rural Technical Service Association, Institute of Cotton Research of CAAS, and Cotton Research Center, Shandong Academy of Agricultural Sciences). Seeds were surface-sterilized by soaking in 15% H_2_O_2_ for 15 min, then rinsed with tap water. Two soaking experimental treatments were designed. Experiment 1: Seeds of 10 cotton cultivars were soaked in deionised water (Control) or Mepiquat chloride (MC, purity 97%, Hebei Guoxin ahadzi Biological Technology Co., Ltd. Hejian, Hebei, China) solution (400 mg/L) at 30 °C for 12 h; Experiment 2: Seeds of K638 were soaked in deionised water (Control) or different concentrations of MC solution (100, 200, and 400 mg/L) at 30 °C for 12 h.

Seeds were germinated between two wetted germination papers (25 cm × 38 cm, Cat.No OP1015, Hoffman Manufacturing Inc., Albany, OR 97321, USA) with a backboard for support. Seven seeds were arranged at 6 cm distance from the top of germination papers with an interval of 2.5 cm. Three replicate germination boards (a total of 21 seeds) per cultivar. The germination boards were placed vertically in the germination tank with tap water to ensure germination papers retained moisture during the experiment process. The germination tank was placed at 25 °C in darkness to promote germination. Once the seedlings grew beyond the top of the germination paper and unshelled the seed coat naturally, the tanks were removed to a customized growth room with 10 h light/14 h dark at (25 ± 0.5/20 ± 0.5)°C, 40–60% relative humidity, and (400 ± 30) μmol m^− 2^ s^− 1^ photosynthetically active radiation.

Images of the cotton seedlings on the germination paper were acquired at 12 DAT (days after soaking seed treatment) for Experiment 1, and were acquired at 6, 8, 10, 12, and 14 DAT for Experiment 2. The number of lateral roots were quantitatively calculated based on these images.

### Dynamic monitoring of root growth

The dynamic effects of MC on root growth were monitored by using the customized high-throughput robotic platform RhizoChamber-Monitor [42]. Cotton seeds of K638 were pretreated by soaking in 400 mg/L MC solution at 30 °C for 12 h, then rinsed with tap water and germinated in silver sand bed (20 cm depth) for 3–4 days until emergence. Before transplanting, the sand was washed away carefully. Thirty-two seedlings per treatment were selected for transplanting. The seedlings were put carefully in the rhizoboxes [42]. The plants were irrigated at 1 h interval with 50 ± 3 mL solution. Nutrient concentrations (mM) were: 2.5 Ca(NO_3_)_2_, 1 MgSO_4_, 0.5 (NH_4_)H_2_PO_4_, 2 × 10^− 4^ CuSO_4_, 1 × 10^− 3^ ZnSO_4_, 0.1 FeNaEDTA, 2 × 10^− 2^ H_3_BO_3_, 5 × 10^− 6^ (NH_4_)_6_Mo_7_O_24_, 1 × 10^− 3^ MnSO_4_, and 0.1 mM K_2_SO_4_. The experiment was carried out in a customized growth room.

The root system of cotton seedlings were monitored for 12 days after transplanting (15 days after soaking treatment). Nine similar plants according to their root morphometry and the image quality were selected for each treatment. A customized image processing software, implemented in Matlab [42], was used to automatically extract the basic root-growth traits, including the total root area, total root length, primary root length, lateral roots length, and lateral root number. The lateral roots number along the different sections of primary root were extracted semi-automatically by using this software. Detailed information of the RhizoChamber-Monitor system and the image processing software were given by Wu et al. [42].

### RNA extraction, cDNA library preparation, and sequencing for RNA-Seq

Seeds of K638 were soaked in deionised water (Control) or 400 mg/L MC solution at 30 °C for 12 h. Seedlings were then germinated using germination paper rolls and were placed at 25 °C in darkness in a growth chamber. Seeds were germinated about 36 h after soaking seeds. The roots of -soaking-seed for 12, 48, and 72 h were subjected to RNA-seq analyses. The root tip region (0 to 4 mm from the root tip) and root middle region (4 to 20–40 mm from the root tip) were sampled separately, except for 12 h because the root was too short to separate. After harvest, samples were immediately frozen in liquid nitrogen and stored at − 80°Cfor RNA isolation. Three independent biological replicates were analyzed for each treatment. Around 30 roots per replicate were collected for sequencing.

Total RNA was extracted with the RNAprep Pure Plant Kit. RNA purity was checked using the NanoPhotometer spectrophotometer (IMPLEN, CA, USA). RNA integrity and quality were assessed by the Agilent RNA 6000 Nano Chip in the Agilent 2100 Bioanalyzer (Agilent Technologies, Santa Clara, CA, USA).

Two μg total RNA per sample was used as input material for the mRNA sample preparations. Sequencing libraries were generated using NEBNext UltraTM RNA Library Prep Kit for Illumina (NEB, USA) following manufacturer’s recommendations and index codes were added to attribute sequences to each sample.

The clustering of the index-coded samples was performed on a cBot Cluster Generation System using TruSeq PE Cluster Kit v4-cBot-HS (Illumina) according to the manufacturer’s instructions. After cluster generation, the library preparations were sequenced on an Illumina Hiseq 4000 platform and paired-end 150 bp reads were generated.

### Statistical analysis of RNA-Seq

#### Quality control

Raw data (raw reads) of fastq format were firstly processed through in-house perl scripts. In this step, clean data (clean reads) were obtained by removing reads containing adapter and ploy-N, and low quality reads from raw data. At the same time, Q20, Q30, GC-content and sequence duplication level of the clean data were calculated. All the downstream analyses were based on clean data with high quality.

Quantification of gene expression level.

Gene expression was normalized as FPKM (fragments per kilobase of exon model per million mapped reads) values [70]. HTSeq v0.5.3 (EMBL, Heidelberg, Germany) was used to count the reads numbers mapped to each gene. FPKM > 1 was used as the threshold to determine whether the gene was expressed in subsequent analysis.

Sample relationships were analyzed by a Principal component analysis (PCA) and hierarchical clustering. PCA was conducted by using the prcomp function in R with default settings. Hierarchical clustering of all samples was generated based on Pearson correlations. Euclidean algorithm-based K-means clustering was performed to generate the expression clusters of gene expression dynamics along three time points using the OmicShare tools (www.omicshare.com/ tools).

#### Differential expression analysis

Differential expression analysis of each two replicates was performed using the DESeq R package [71]. DESeq provide statistical routines for determining differential expression in digital gene expression data using a model based on the negative binomial distribution. The resulting *P* values were adjusted using the Benjamini and Hochberg’s approach for controlling the false discovery rate. Genes with an adjusted *P*-value < 0.05 were assigned as differentially expressed.

GO and KEGG enrichment analysis of differentially expressed genes (DEGs).

Gene Ontology (GO) enrichment analysis of DEGs was implemented by the GOseq R packages based Wallenius non-central hyper-geometric distribution [72], which can adjust for gene length bias in DEGs. KEGG [73], a database resource for understanding high-level functions and utilities of the biological system, such as the cell, the organism, and the ecosystem at molecular-level was generated by genome sequencing and other high-throughput experimental technologies (http://www.genome.jp/kegg/). We used KOBAS [74] software to test the statistical enrichment of DEGs in KEGG pathways.

### Quantitative real-time PCR (qRT-PCR) analysis

cDNA synthesis was performed with Superscript II reverse transcriptase (Invitrogen) according to the manufacturer’s instructions. qRT-PCR was performed using SYBR Green Master Mix. The reaction volume was 15 μl which contained 1.5 μl of diluted cDNA, 0.3 μl of ROX reference dye, 0.3 μl of each 10 μM forward primer and reverse primer, and 7.5 μl SYBR Premier Ex Taq mix (Takara, Japan). PCR amplification was performed using two-step cycling conditions of 95 °C for 30 s, followed by 40 cycles of 95 °C for 5 s and 60 °C for 35 s. The levels of each gene transcript were calculated relative to its corresponding untreated control. Fold-changes of RNA transcripts were calculated by C_T_ methods [75]. Twelve reference genes were selected for qRT-PCR analysis. The primers for each reference gene were listed in Additional file 5: Table S5.

### Endogenous hormonal level measurement

Plant hormones, IAA, GA, and ABA in roots at five DAT, were extracted and purified according to the protocol described in Yang et al. [76]. Segments of approximately 2 cm root were cut and washed with de-ionized water. About 0.5 g of fresh roots was homogenized in 2 ml 80% methanol and stored at − 20 °C for 48 h. The extract was centrifuged at 4000 g for 15 min at 4 °C, and then the supernatant was passed through C18 Sep-Pak cartridges (Waters Corp., Millford, MA, USA). The sediments were re-suspended with 10 ml of 100% (v/v) methanol and 10 ml of ether. Afterwards, the eluate was dried down by pure N2 at 20 °C, and then stored at − 40 °C. The concentration of plant hormones was determined by ELISA technique following the protocol described in Zhao et al. [77]. Endogenous free IAA, GA, and ABA were calculated according to Weiler et al. [78].

## Supplementary information

**Additional file 1: Table S1.** Summary of sequence assembly after Illumina sequencing.

**Additional file 2: Table S2.** Statistics of genes in different expression-level interval.

**Additional file 3: Table S3.** GO annotation enrichment analysis for differentially expressed genes.

**Additional file 4: Table S4.** The list of ABA-, GA-, and ethylene-related differentially expressed genes

**Additional file 5: Table S5.** The primers for each reference gene for qRT-PCR analysis.

**Additional file 6: Figure S1.** The relationship of transcriptome samples. A. Principal component analysis (PCA) of Control and MC RNA-Seq samples at three time points after MC treatment; B. Hierarchical clustering of the RNA-Seq samples based on Pearson correlation. Height indicates the degree of variance of the y-axis. Cotton seeds of K638 were treated with deionised water (Control) or 400 mg/L MC for 12 h. The roots at 12, 48, and 72 h after treatment were subjected to RNA-seq analyses. “R” indicates the whole root, “M” indicates the root middle region (4 to 20–40 mm from the root tip), and “T” indicates the root tip region (0 to 4 mm from the root tip).

**Additional file 7: Figure S2.** The functional annotation and GO enrichment of the down-regulated and up-regulated DEGs at each time point for root middle region and root tip.

**Additional file 8: Figure S3.** Statistical analyses of functional enrichments by KEGG pathways. Values in boxes are the number of enriched genes, values in boxes and in brackets are *P*-value (0 stands for *P < 0.01*).

**Additional file 9: Figure S4.** Up-regulated and down-regulated hormone-related DEGs upon MC treatment. Cotton seeds of K638 were treated with deionised water (Control) or 400 mg/L MC soaking-seed for 12 h. The DEGs were collected with FDR < 5%.

**Additional file 10: Figure S5.** Up-regulated and down-regulated cell cycle/division-related DEGs upon MC treatment that belonged to different genes family, including *CDC, CDK, Cyclin, KRP, CYP, FKBP* and *SMC*. Cotton seeds of K638 were treated with deionised water (Control) or 400 mg/L MC for 12 h. The DEGs were collected at 72 h after MC treatment in the root middle region (FDR < 5%).

**Additional file 11: Figure S6.** The relative expression pattern of auxin- and cell cycle-related genes. A. The dynamics expression of auxin biosynthesis and transport genes in Control and MC. B. Relative transcript levels of genes corresponding to A by qRT-PCR. C. The relative expression of auxin- and cell cycle-related genes in Control and MC. D. Relative transcript levels of genes corresponding to C by qRT-PCR.

## Data Availability

The datasets supporting the conclusions of this article are available in https://github.com/wuqiangithub/MC_Root_RNA-seq.

## Abbreviations

BZL/PL: Branching zone length /Primary root length
DAT: Days after soaking seed treatment
DEGs: Differentially expressed genes
FPKM: Fragments per kilobase of exon model per million mapped reads
MC: Mepiquat chloride
PCA: Principal component analysis
