# Supplementary figures and images for: Mepiquat chloride promotes cotton lateral root formation by modulating plant hormone homeostasis
Source: BMC Plant Biol. 2019 Dec 21;19:573. doi: 10.1186/s12870-019-2176-1 (PMC6925410; doi:10.1186/s12870-019-2176-1)

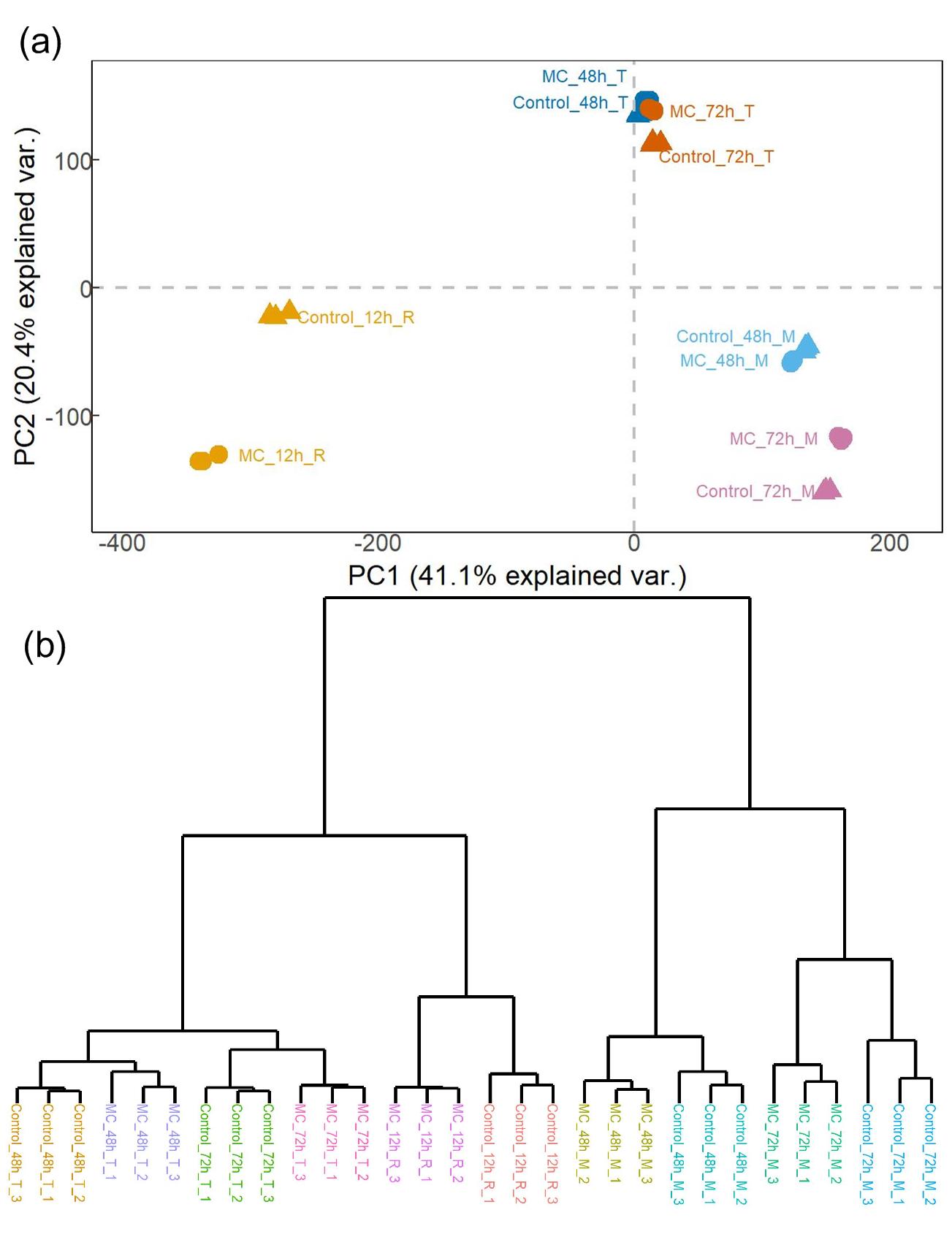

Supplement: Supplementary file 6 — Additional file 6: Figure S1. The relationship of transcriptome samples. A. Principal component analysis (PCA) of Control and MC RNA-Seq samples at three time points after MC treatment; B. Hierarchical clustering of the RNA-Seq samples based on Pearson correlation. Height indicates the degree of variance of the y-axis. Cotton seeds of K638 were treated with deionised water (Control) or 400 mg/L MC for 12 h. The roots at 12, 48, and 72 h after treatment were subjected to RNA-seq analyses. “R” indicates the whole root, “M” indicates the root middle region (4 to 20–40 mm from the root tip), and “T” indicates the root tip region (0 to 4 mm from the root tip). [file 12870_2019_2176_MOESM6_ESM.jpg]

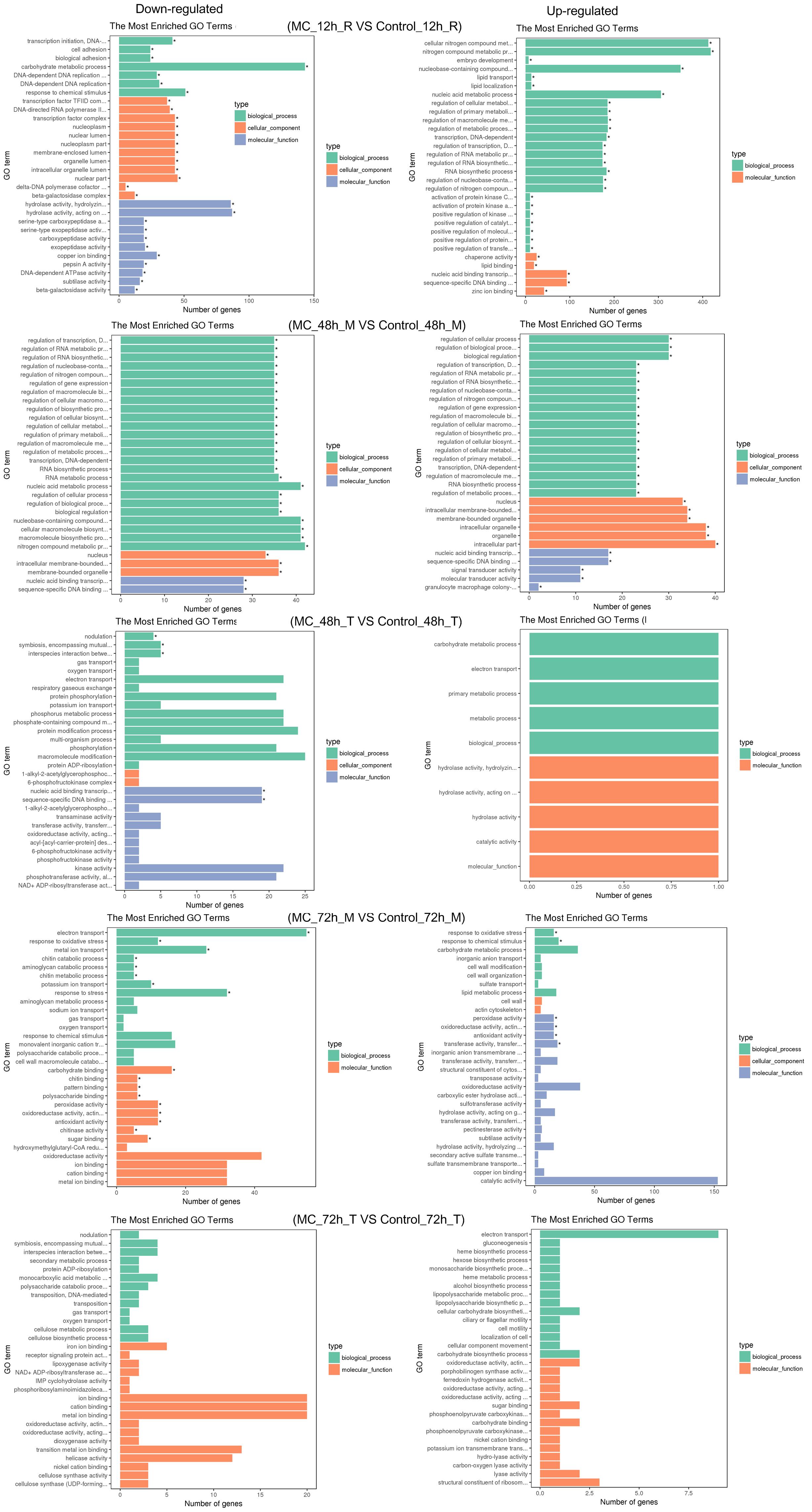

Supplement: Supplementary file 7 — Additional file 7: Figure S2. The functional annotation and GO enrichment of the down-regulated and up-regulated DEGs at each time point for root middle region and root tip. [file 12870_2019_2176_MOESM7_ESM.jpg]

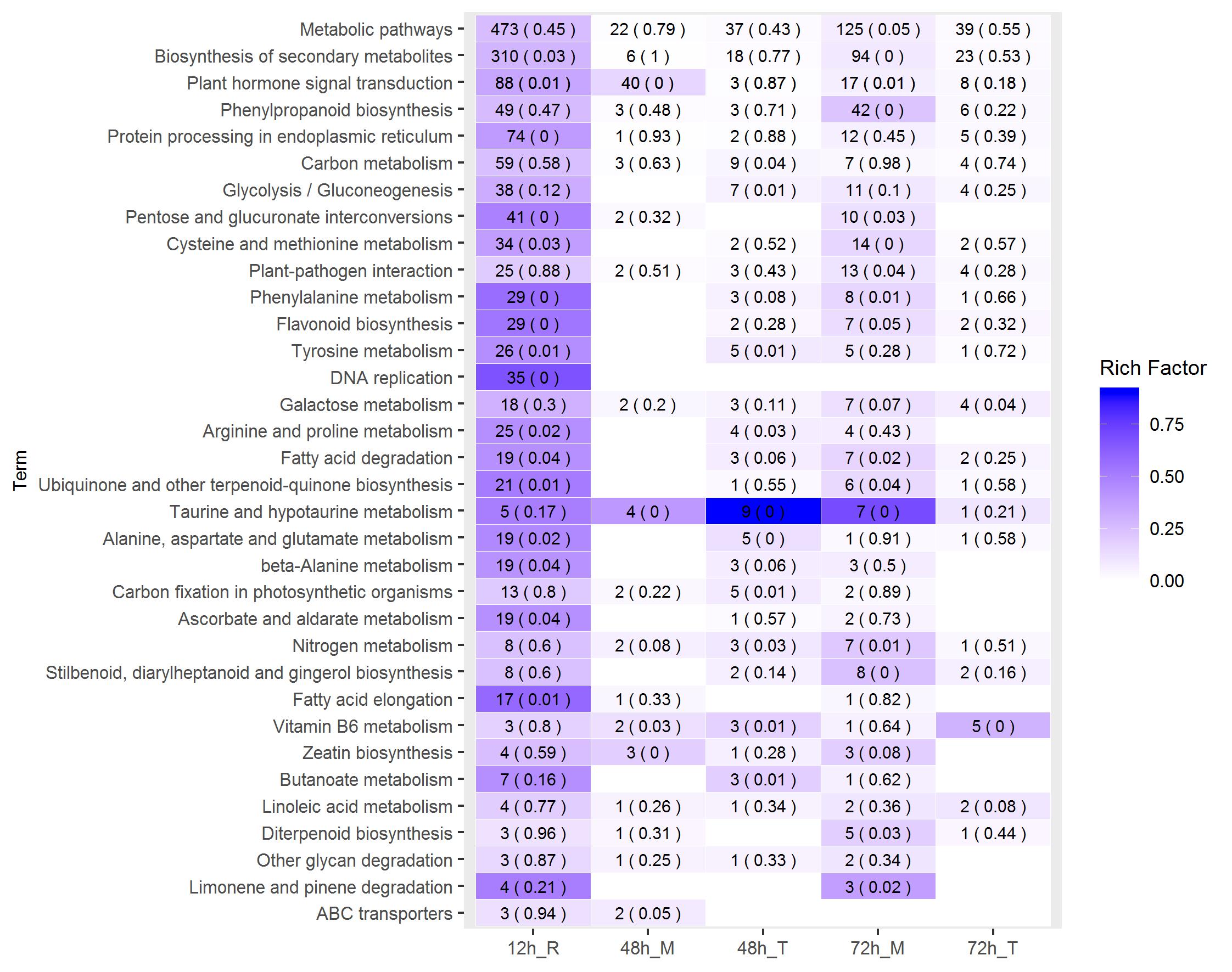

Supplement: Supplementary file 8 — Additional file 8: Figure S3. Statistical analyses of functional enrichments by KEGG pathways. Values in boxes are the number of enriched genes, values in boxes and in brackets are P-value (0 stands for P < 0.01). [file 12870_2019_2176_MOESM8_ESM.jpg]

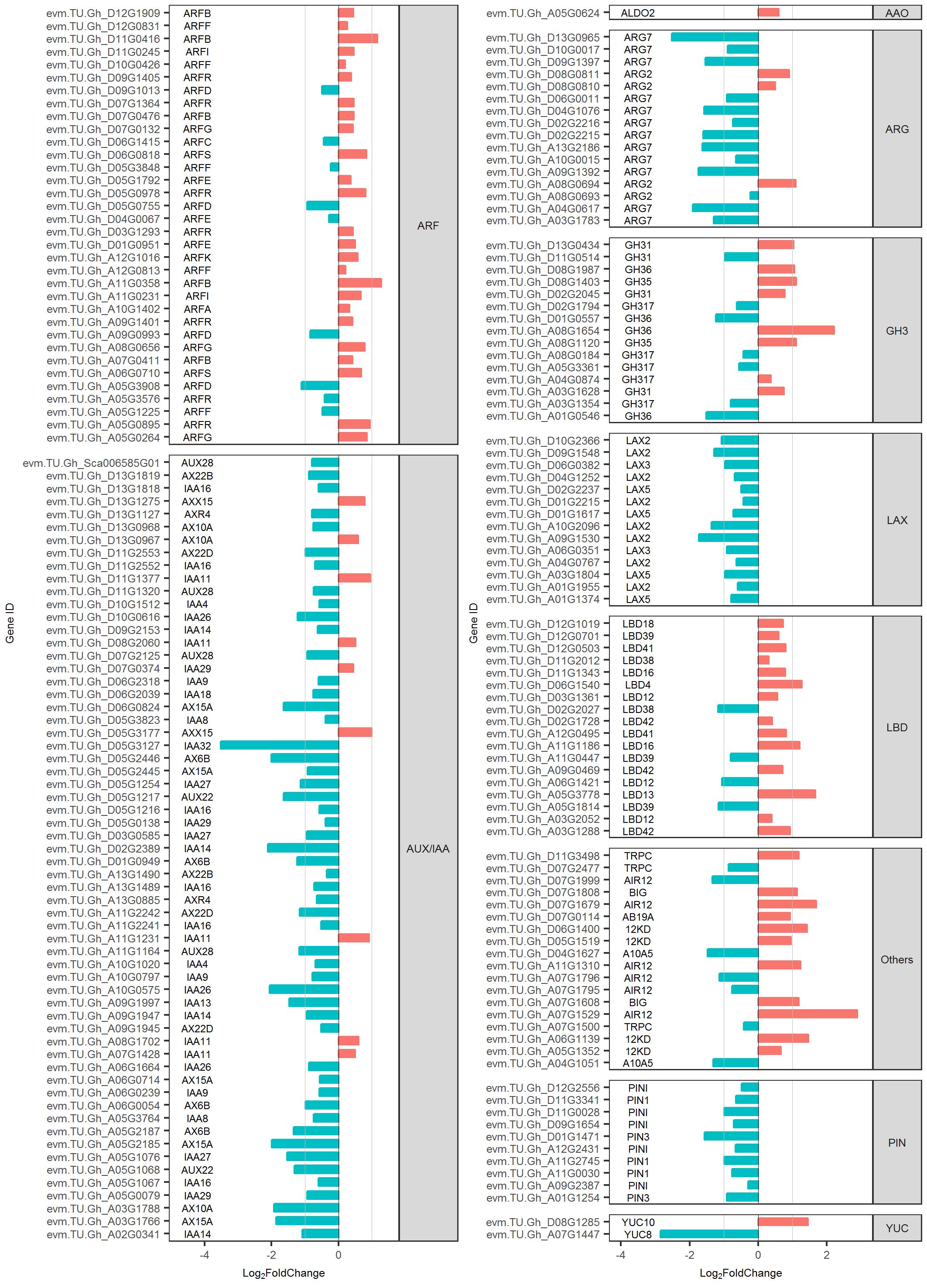

Supplement: Supplementary file 9 — Additional file 9: Figure S4. Up-regulated and down-regulated hormone-related DEGs upon MC treatment. Cotton seeds of K638 were treated with deionised water (Control) or 400 mg/L MC soaking-seed for 12 h. The DEGs were collected with FDR < 5%. [file 12870_2019_2176_MOESM9_ESM.jpg]

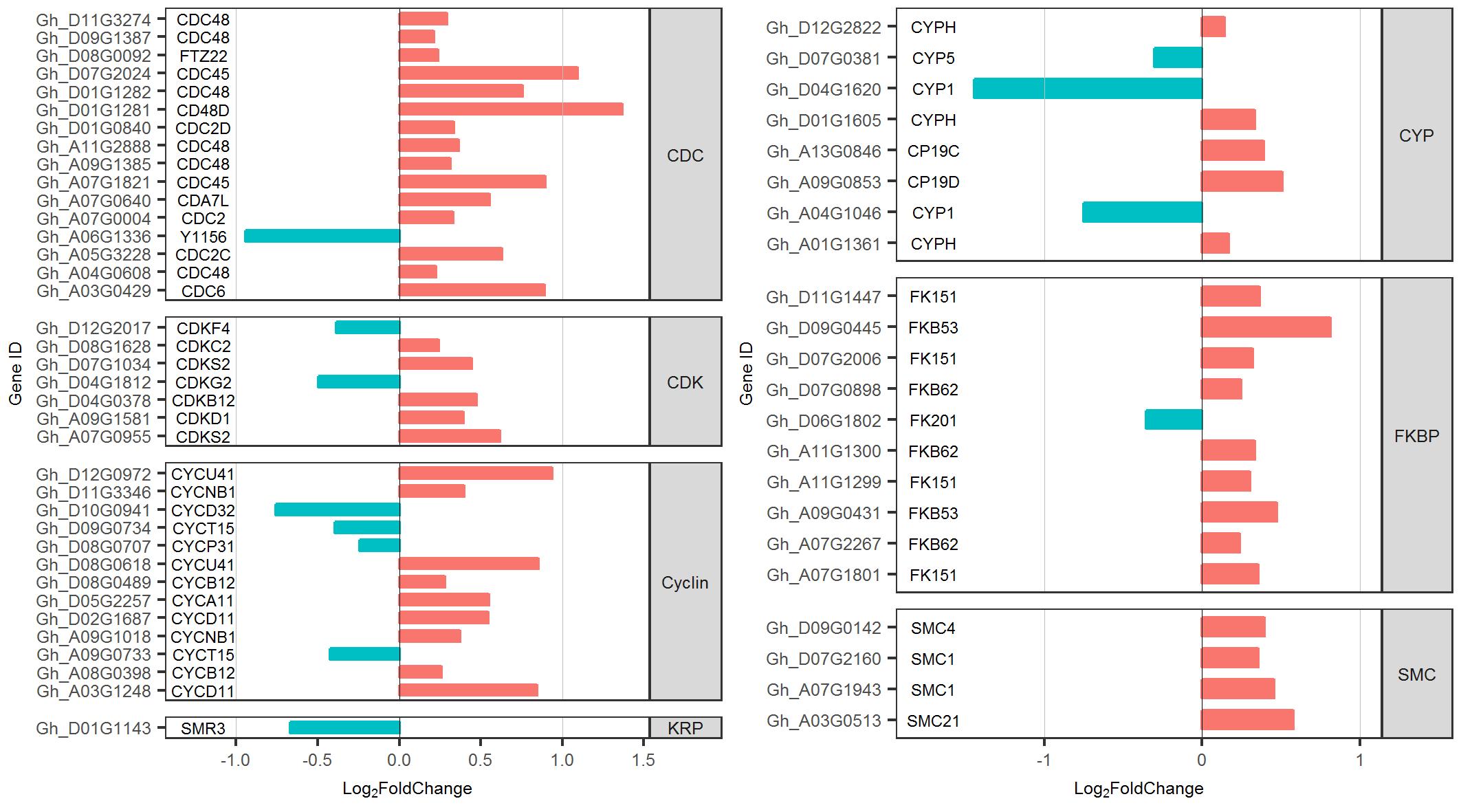

Supplement: Supplementary file 10 — Additional file 10: Figure S5. Up-regulated and down-regulated cell cycle/division-related DEGs upon MC treatment that belonged to different genes family, including CDC, CDK, Cyclin, KRP, CYP, FKBP and SMC. Cotton seeds of K638 were treated with deionised water (Control) or 400 mg/L MC for 12 h. The DEGs were collected at 72 h after MC treatment in the root middle region (FDR < 5%). [file 12870_2019_2176_MOESM10_ESM.jpg]

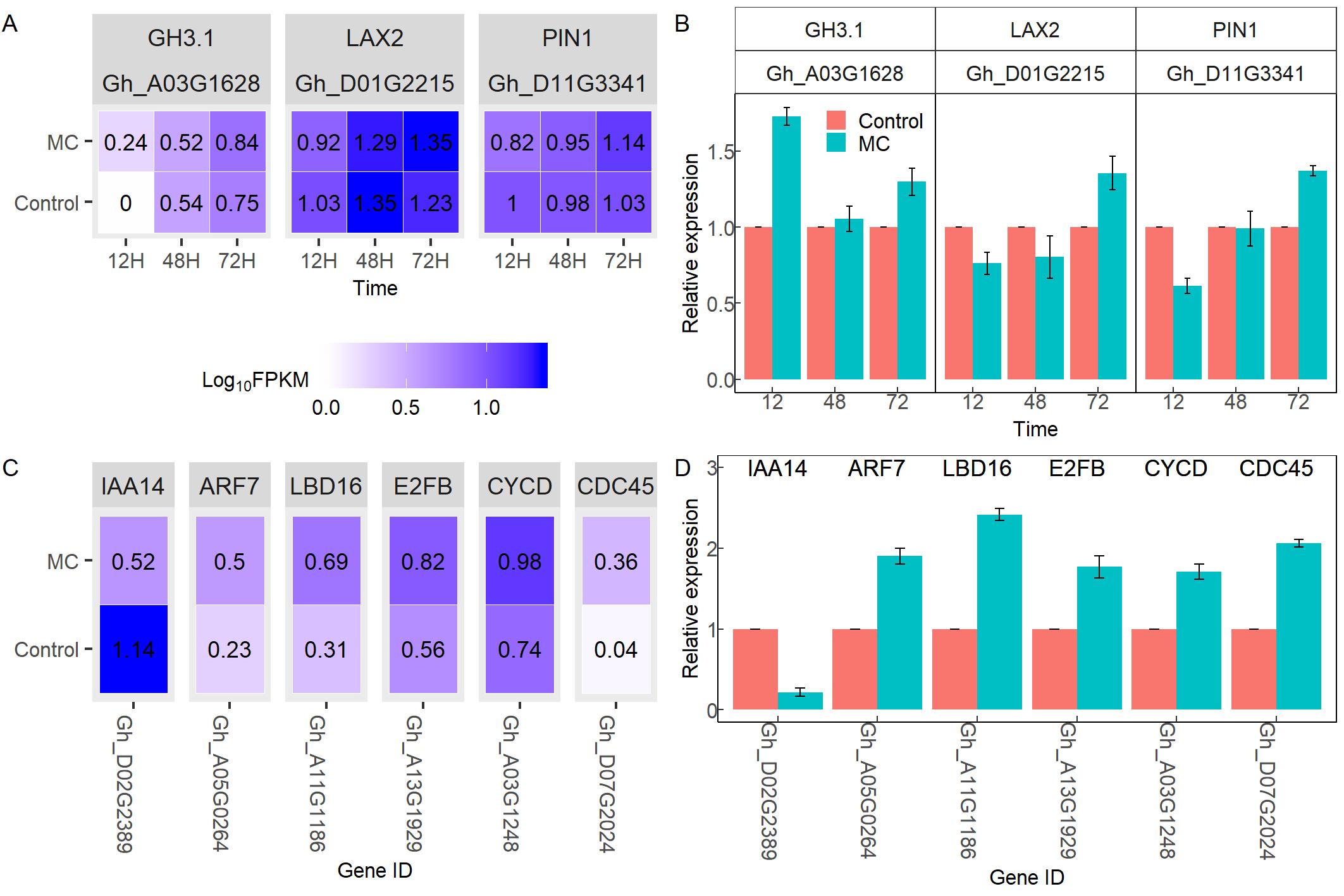

Supplement: Supplementary file 11 — Additional file 11: Figure S6. The relative expression pattern of auxin- and cell cycle-related genes. A. The dynamics expression of auxin biosynthesis and transport genes in Control and MC. B. Relative transcript levels of genes corresponding to A by qRT-PCR. C. The relative expression of auxin- and cell cycle-related genes in Control and MC. D. Relative transcript levels of genes corresponding to C by qRT-PCR. [file 12870_2019_2176_MOESM11_ESM.jpg]
